# The Emergency Medical Team Initiative in the Western Pacific Region

**DOI:** 10.5365/wpsar.2023.14.6.1161

**Published:** 2024-10-08

**Authors:** Natasha Mamea, Atoa Glenn Fatupaito, Md Anuar bin Abd Samad, Mahmood,, Ridzuan Bin Dato' Mohd Isa, Gaafar Uherbelau, Esther Muña, Shalimar Abdullah, Masniza Mustaffa

**Affiliations:** a Ministry of Health, Apia, Samoa.; b Ministry of Health, Putrajaya, Malaysia.; c Minister of Health, Ministry of Health and Human Services, Koror, Palau.; d CEO, Commonwealth Healthcare Corporation, Saipan, Northern Mariana Islands, United States of America.; e Mercy Malaysia Emergency Medical Team, Kuala Lumpur, Malaysia.

Emergency medical teams (EMTs) are often among the first responders when disease outbreaks or disasters strike. As self-sufficient, deployable field hospitals and clinics staffed by trained team members and equipped with pre-positioned clinical and non-clinical supplies (known as “EMT cache”), EMTs are primed to deliver high-quality clinical care and public health response to affected populations in the most remote locations and austere conditions. Based on WHO’s *Classification and minimum standards for emergency medical teams* (2021), (*1*) EMTs are committed to excellence in clinical care and apply a principled approach to health emergency response, which includes working under national leadership in the affected country or area and not adding any burden to local responders. EMTs’ clinical scope ranges from routine outpatient care to highly specialized surgery and intensive care, and they respond to a wide range of emergencies, including infectious disease outbreaks, disasters, conflicts and other human-induced events.

This Special Edition of WHO’s *Western Pacific Surveillance and Response* (WPSAR) journal provides a range of insights into how EMTs are developed and maintained, how team members are trained, how EMTs provide care in response to a wide range of emergencies, and some of the challenges they face in these efforts. It includes articles written by emergency responders from a range of country contexts, as well as by WHO experts supporting EMT development and coordination in emergencies. It was developed with the explicit goal of expanding research and documentation related to the work of EMTs, as articulated in WHO’s EMT 2030 strategy. (*2*)

In 2022, during the fifth EMT Global Meeting hosted in Armenia, a new Western Pacific Regional EMT Chair Group was appointed for the calendar year 2023. The 2023 Chair Group membership – composed of representatives of health ministries and departments from Malaysia, the Commonwealth of the Northern Mariana Islands (United States of America), Palau and Samoa, as well as the nongovernmental organization (NGO) MERCY Malaysia – was appointed to reflect the Region’s extensive and diverse network of national and international EMTs. The Region’s EMTs, which range from very small mobile teams in several Pacific island countries and areas (PICs) to much larger field hospitals, as well as specialized care teams that can deploy to existing health facilities, (*3*, *4*) mirror the demographic diversity of the Region itself, with some of the smallest and largest countries in the world, both in terms of landmass and population.

By the end of 2023, nearly every country in the Region had established at least one EMT or had become engaged with the EMT Initiative. The Region hosts 12 of the world’s 40 classified international EMTs (as of late 2023), which are teams that have undergone mentorship and an external quality assurance process to ensure quality and predictability when they deploy internationally in response to emergencies. Teams based in larger, higher-income countries such as Australia, China and Japan are well established with highly advanced capabilities and have responded to health emergencies around the world while also serving as mentors to emerging EMTs. At the same time, 13 PICs have developed or are in the process of establishing EMTs, including one internationally classified team in Fiji. (*3*) Other countries in the Region, including Mongolia, the Philippines, Singapore and countries in the Greater Mekong Subregion, are also establishing EMTs, with some working to achieve international EMT classification in the near future. The Region’s EMT network is also enriched by multiple teams managed by NGOs in Australia, Japan, Malaysia and New Zealand, which are actively engaged in health emergency response within the Region and around the world.

Western Pacific EMTs are largely managed by ministries of health, but many involve other government agencies, such as the armed forces, civil defence forces, fire and rescue services and national disaster management offices. Western Pacific EMT rosters include thousands of individual team members, with an enormous range of clinical and non-clinical expertise and extensive experience in complex health emergency settings.

EMTs are routinely called upon to care for affected communities in their greatest moments of need. (*3*-*6*) Based upon the requirements of the International Health Regulations (IHR 2005) and recommendations from the Asia Pacific Health Security Action Framework (APHSAF), significant work on EMT development has been carried out across the Region, which has improved country preparedness and operational readiness and strengthened regional surge response capacities. (*7*, *8*) Recent EMT successes include the deployment of the Tonga Emergency Medical Assistance Team (TEMAT) to the Ha’apai island group following the January 2022 volcanic eruption and tsunami, (*4*) multiple deployments of the Philippines Emergency Medical Assistance Team (PEMAT) in response to domestic disasters, (*9*) as well as many larger national and international EMT deployments in response to the COVID-19 pandemic across the Region.

While many EMTs in the Region have been working towards strengthening their readiness for deployment at home, others have also actively engaged in health emergency response beyond their borders. In 2023, the Japan Disaster Relief (JDR) EMT and PEMAT responded to earthquakes in Türkiye and Syrian Arab Republic, while the Fiji Emergency Medical Assistance Team (FEMAT) deployed to Vanuatu in response to Tropical Cyclone Lola. In 2020–2022, the Australia Medical Assistance Team (AUSMAT) and the New Zealand Medical Assistance Team (NZMAT) supported COVID-19 response efforts in multiple PICs.

To support EMT development in the Region, WHO’s Regional Office for the Western Pacific procured and delivered EMT cache to national EMTs across the Pacific, as well as to teams in Cambodia, Lao People's Democratic Republic, Mongolia and the Philippines throughout 2022 and 2023. (*3*, *10*) WHO and its donor partners also supported national team members’ training in 14 countries across the Region in 2023 alone. (*3*, *11*)

In the years ahead, further growth and development of EMTs in the Region is planned. More than 10 Western Pacific EMTs are currently working towards WHO EMT classification, with several teams aiming to achieve this in 2024. Meanwhile, several existing EMTs are working towards reclassification in 2024, which is required every 5 years following initial classification. National EMT development will continue through activities such as team member trainings and simulation exercises and faculty/mentor exchanges, all of which contribute to the sharing of experiences and lessons across countries, areas and teams. The common cache procured by WHO for Pacific EMTs and the implementation of a consistent training approach will serve to strengthen EMT interoperability going forward, in line with the EMT 2030 strategy. (*2*, *10*)

## Working towards EMT 2030

The 2023 Western Pacific Region EMT Chair Group has endorsed the goals outlined in the Global EMT Network’s EMT 2030 strategy, which states, “[I]n times of crisis, national EMTs are in the best position to provide immediate assistance. Strengthening both national and international EMTs and increasing interoperability with other rapid response capacities are essential components of a country's emergency preparedness and response to save lives, improve health and serve the most vulnerable and in need.” (*2*) The Western Pacific Region is already making significant progress in achieving the targets outlined in the EMT 2030 strategy, which align with World Health Assembly document WHA75/20 (Strengthening the global architecture for health emergency preparedness, response and resilience), WHO’s Triple Billion targets, and the United Nation’s Sustainable Development Goals. (*12*-*15*)

In the years ahead, EMTs across the Region will continue to expand partnerships, strengthen local leadership, support national EMT focal points, and further refine the regional EMT governance structure. The Western Pacific Regional EMT Chair Group will continue to provide guidance to EMTs and to the WHO Secretariat, and will advocate for sustainable, institutionally grounded rapid response capabilities and financial investments in EMT development and operations. The Western Pacific Region has a long history of partnership and knowledge sharing and will continue to build on this tradition to enhance capacities to respond to emergencies nationally, regionally and globally.

EMTs contributing to regional health security in the Western Pacific

The newly published APHSAF identified several priorities for the Region, including “to protect the health and well-being of communities in the Asia-Pacific region by strengthening, maintaining and enhancing multisectoral health security capacities and systems to prevent, prepare, respond and increase resilience to multi-hazard public health emergencies.” (*8*) The previous, current and planned EMT work across the Region is aligned with this vision, aiming to create “an Asia-Pacific region that is prepared for and resilient to public health emergencies through collective action and that contributes to global health security.”

The Western Pacific EMT Chair Group is committed to supporting and strengthening health emergency response capabilities in the WHO Western Pacific Region to ensure a more secure health future for peoples of the Region and beyond.

## References

[1] Classification and minimum standards for emergency medical teams. Geneva: World Health Organization; 2021. Available from: https://iris.who.int/handle/10665/341857, accessed 13 March 2024.

[2] Emergency medical teams 2030 strategy. Geneva: World Health Organization; 2023. Available from: https://iris.who.int/handle/10665/372867, accessed 13 March 2024.

[3] Casey ST, Noste E, Cook AT, Larsen J-E, Cowie S, Ferguson MM, et al. Localizing health emergency preparedness and response: emergency medical team development and operations in Pacific island countries and areas. Western Pac Surveill Response J. 2023;14(6 Spec edition):1–4. doi:10.5365/wpsar.2023.14.6.1021 pmid:37969417. 10.5365/wpsar.2023.14.6.1021 PMC10645487 37969417

[4] Sifa S, Fusi SKF, Casey ST, Poloniati P, Tavo K, Setoya Y, ‘Akauola ‘A. Tonga national emergency medical team response to the 2022 Hunga Tonga-Hunga Ha’apai volcanic eruption and tsunami: the first deployment of the Tonga Emergency Medical Assistance Team (TEMAT). Western Pac Surveill Response J. 2023;14(6 Spec edition):1–6. doi:10.5365/wpsar.2023.14.6.1026 pmid:37969419. 10.5365/wpsar.2023.14.6.1026 PMC10645485 37969419

[5] Curtis SJ, Trewin A, McDermott K, Were K, Walczynski T, Notaras L, et al. An outdoor hotel quarantine facility model in Australia: best practice with optimal outcomes. Aust N Z J Public Health. 2022 Oct;46(5):633–9. 10.1111/1753-6405.13275 35797090 PMC9349389

[6] Casey ST, Mamea-Maa NA, Nofoaiga M, Martin B, Henshall KA, Fidow M, et al. The roles of emergency medical teams in response to Samoa’s 2019 measles outbreak. Western Pac Surveill Response J. 2024;14(6 Spec edition):1–7. doi:10.5365/wpsar.2023.14.6.1031 pmid:38745981. 10.5365/wpsar.2023.14.6.1031 PMC11089276 38745981

[7] International health regulations (2005), 3rd ed. Geneva: World Health Organization; 2016. Available from: https://iris.who.int/handle/10665/246107, accessed 8 May 2024.

[8] Asia Pacific health security action framework. Manila: WHO Regional Office for the Western Pacific; 2024. Available from: https://iris.who.int/handle/10665/377083, accessed 21 July 2024.

[9] Philippine emergency medical teams respond to Taal Volcano eruption. Manila: WHO Representative Office for the Philippines; 2020. Available from: https://iris.who.int/handle/10665/377083, accessed 22 July 2024.

[10] Beauchemin P-Y, Chandler DR, Noste EE, Larsen J-E, Cook AT, Casey ST. Development and procurement of a national emergency medical team (EMT) cache for Pacific island countries. Prehosp Disaster Med. 2022;37 S2:s89. 10.1017/S1049023X22001881 36518001

[11] Noste EE, Cook AT, Larsen JE, Cowie S, Casey ST. Tailoring a national emergency medical team training package for Pacific island countries and areas. Western Pac Surveill Response J. 2023;14(6 Spec edition):1–6. doi:10.5365/wpsar.2023.14.6.1033 pmid:38298251. 10.5365/wpsar.2023.14.6.1033 PMC10824629 38298251

[12] Seventy-fifth World Health Assembly. Report by the Director-General. Strengthening the global architecture for health emergency preparedness, response and resilience. Geneva: World Health Organization; 2022 (A75/20). Available from: https://apps.who.int/gb/ebwha/pdf_files/WHA75/A75_20-en.pdf, accessed 13 March 2024.

[13] Strengthening health emergency prevention, preparedness, response and resilience. Geneva: World Health Organization; 2023. Available from: https://cdn.who.int/media/docs/default-source/emergency-preparedness/who_hepr_wha2023−21051248b.pdf?sfvrsn=a82abdf4_3&download=true, accessed 13 March 2024.

[14] Resolution WHA71. 1. In: Seventy-first World Health Assembly. Thirteenth general programme of work 2019–2023. Geneva: World Health Organization; 2018. Available from: https://iris.who.int/handle/10665/279451, accessed 13 March 2024.

[15] Transforming our world: the 2030 agenda for sustainable development. A/Res/70/1. New York (NY): United Nations; 2015. Available from: https://sdgs.un.org/sites/default/files/publications/21252030%20Agenda%20for%20Sustainable%20Development%20web.pdf, accessed 13 March 2024.

